# Multiple transcriptome analyses reveal mouse testis developmental dynamics

**DOI:** 10.1186/s12864-024-10298-y

**Published:** 2024-04-22

**Authors:** Anqi Chen, Chaoneng Ji, Chengtao Li, Beate Brand-Saberi, Suhua Zhang

**Affiliations:** 1 Institute of Forensic Science, Fudan University, 200032 Shanghai, China; 2 State Key Laboratory of Genetic Engineering, School of Life Sciences, Fudan University, 200438 Shanghai, China; 3 Shanghai Key Laboratory of Forensic Medicine, Shanghai Forensic Service Platform, Ministry of Justice, Academy of Forensic Science, 200063 Shanghai, China; 4 Department of Anatomy and Molecular Embryology, Institute of Anatomy, Medical Faculty, Ruhr University Bochum, 44801 Bochum, Germany

**Keywords:** Testis development, Whole transcriptome sequencing, RNA dynamics

## Abstract

The testes are the organs of gamete production and testosterone synthesis. Up to date, no model system is available for mammalian testicular development, and only few studies have characterized the mouse testis transcriptome from no more than three postnatal ages. To describe the transcriptome landscape of the developing mouse testis and identify the potential molecular mechanisms underlying testis maturation, we examined multiple RNA-seq data of mouse testes from 3-week-old (puberty) to 11-week-old (adult). Sperm cells appeared as expected in 5-week-old mouse testis, suggesting the proper sample collection. The principal components analysis revealed the genes from 3w to 4w clustered away from other timepoints, indicating they may be the important nodes for testicular development. The pairwise comparisons at two adjacent timepoints identified 7,612 differentially expressed genes (DEGs), resulting in 58 unique mRNA expression patterns. Enrichment analysis identified functions in tissue morphogenesis (3-4w), regulation of peptidase activity (4-5w), spermatogenesis (7-8w), and antigen processing (10-11w), suggesting distinct functions in different developmental periods. 50 hub genes and 10 gene cluster modules were identified in the testis maturation process by protein-protein interaction (PPI) network analysis, and the miRNA-lncRNA-mRNA, miRNA-circRNA-mRNA and miRNA-circRNA-lncRNA-mRNA competing endogenous RNA (ceRNA) networks were constructed. The results suggest that testis maturation is a complex developmental process modulated by various molecules, and that some potential RNA-RNA interactions may be involved in specific developmental stages. In summary, this study provides an update on the molecular basis of testis development, which may help to understand the molecular mechanisms of mouse testis development and provide guidance for mouse reproduction.

**Supplementary Information:**

The online version contains supplementary material available at 10.1186/s12864-024-10298-y.

## Introduction

According to the World Health Organization (WHO), more than 186 million people suffer from infertility [1], with half of fertility problems caused by men [2]. Several studies have attempted to analyze the mechanisms and pathways underlying male infertility, but male-factor infertility remains unexplainable [2–5]. In vitro immortalized cell lines are the first choice for studying cell functions [6], but to date no immortal germ cell line has been constructed. Organoids are derived from pluripotent stem cells (PSCs), including embryonic stem cells (ESCs) and induced pluripotent stem cells (iPSCs), are intended to reproduce the morphological structure, physiological function and other characteristics of the source tissue [7]. Recently, Knarston et al. [8] have generated early testis-like cells with the ability to be cultured as an organoid, providing a step forward in human gonadal organoid. However, there are still limitations to be overcome, including lack of patterning, limited maturation and atypical physiology, et al. [9]. It is almost impossible to fully recapitulate all in vitro biochemical cues that drive cell differentiation and 3D tissue assembly at the precise times, places and concentrations that they would occur during embryonic development [10]. As a result, the house mouse (Mus musculus) has become the preferred mammalian model for studying testis development, but the underlying molecular events and mechanisms during mouse testicular development have not been fully elucidated.

Lukassen et al. [11] investigated transcriptional dynamics during spermatogenesis using single-cell RNA sequencing (scRNA-seq), which has provided useful information for marker discovery in different cell populations. It has been reported that the capture rate of scRNA-seq is approximately 6-30% [12], which may result in an incomplete gene expression matrix. Wang et al. [13] found higher noise and more severe gene dropouts when comparing the two representative scRNA-seq platforms. The scRNA-seq may be sufficient to classify a cell type, but not to detect the genes with low expression [14, 15]. Furthermore, most scRNA-seq studies focus only on the mRNA expression profile, neglecting the transcriptomes of non-coding RNA (ncRNA) and small RNA. As ncRNAs and small RNAs have been implicated in male infertility [16], these scRNA-seq datasets may not be representative of testis development. Therefore, a high-throughput, multidimensional RNA profile is expected to be more valuable than unilateral expression research [17]. RNA-seq is considered to be a reliable tool to gain insight into the transcriptome [17–20]. Gong et al. [21] investigated the developing mouse testes at the transcriptional level in pups (6 days postnatal), juveniles (4 weeks old), and adults (10 weeks old), but the study mainly focused on mRNA changes, and the roles that small RNAs may play in development remained unknown. Unlike oocytes, spermatogenesis starts at puberty and continues until death [22]. Therefore, studying the testicular transcriptome at shorter time points may reflect the dynamic transitions during testicular maturation.

To further understand the developmental process in the postnatal period, we performed whole-transcriptome sequencing of mouse testes over eleven consecutive postnatal weeks. Differentially expressed genes (DEGs) were identified. The enrichment analysis on the DEGs predicts the functional process as well as the key pathways during testis development. Subsequently, the networks of differential mRNAs, miRNAs, lncRNAs and circRNAs were built to reveal the functional interaction during testis maturation, and the hub genes associated with testis maturation were screened out.

## Materials and methods

### Animal study

The 3–11 weeks (w) old BL/6J mice (C57BL/6J) mice were purchased from Shanghai Slake Experimental Animal Co., Ltd. (Slake, China), and were raised under standard laboratory conditions with a 12-hour light/dark cycle and free access to food and water. Testes were dissected at the age of 3, 4, 5, 6, 7, 8, 9, 10 and 11weeks of age, respectively. A total of 27 mice (9 time points × 3 mice) were sacrificed for this study, and the harvested tissues were stored at -80 ℃ until use.

### Histological analysis

The testes were dehydrated in gradient ethanol, cleared in xylene, and embedded in paraffin. Embedded tissues were cut into 5 μm sections and stainied with hematoxylin and eosin (H&E) for histological observation by brightfield microscopy.

### RNA extraction, library construction and sequencing

Total RNA was extracted using TRIzol reagent (Invitrogen, USA) according to the manufacturer’s protocols. The rRNA was removed using Ribo-Zero™ rRNA Removal Kit (Human/Mouse/Rat) (Illumina, USA) according to the manufacturer’s recommendations. RNA concentrations were determined using a Qubit 2.0 Fluorometer (Thermo Fisher Scientific, USA). The integrity and the purity of the total RNA were verified by 2% denaturing agarose gels prior to library construction. To minimize the batch effect in sequencing and the variability among individuals, the RNA samples of the same age were pooled together with equal inputs before library construction.

RNA-seq libraries (mRNA, lncRNA and circRNA) were constructed using 10 µg of the total RNA. The purified RNA fractions were fragmented under elevated temperature using divalent cations. The cleaved RNA fractions were reverse transcribed to generate the double-stranded cDNA libraries following the instructions for the mRNA-Seq Sample Preparation Kit (Illumina, USA), and the insert size for the paired-end libraries was approximately 300 bp. The pooled library size was assessed by analysis on the Agilent 2100 BioAnalyzer (Agilent Technologies, USA), and quantified using a Qubit 2.0 Fluorometer (Thermo Fisher Scientific, USA). Each library was paired-end sequenced on an Illumina NovaSeq 6000 (2 × 150 bp read length) according to the manufacturer’s instructions.

Small RNA-seq libraries (miRNA, piRNA and ncRNA) were prepared from 1 µg total RNA input using the TrueSeq Small RNA Sample Prep Kit (Illumina, USA) according to the manufacturer’s instructions. Each library was single-end sequenced using an Illumina NovaSeq 6000 (1 × 150 bp read length) according to the manufacturer’s instructions.

### Quality control and RNAs expression analysis

To ensure that the sequencing data were suitable for further analysis, we used the program FastQC (http://www.bioinformatics.babraham.ac.uk/projects/fastqc/) to check the overall quality of the reads (FASTQ format) for each sample. The adapter sequences, polyA tails, low-quality sequences (reads with a Phred quality score less than 20) were removed using Cutadapt (https://cutadapt.readthedocs.io/en/stable/). For small RNA-seq data, the sequences with less than 18 nucleotides or more than 36 nucleotides were also discarded from the raw sequence data.

Trimmed clean reads were aligned to the mouse genome (Mus_musculus.GRCm39) using TopHat2 [23] and Bowtie2 [24] and the number of reads uniquely mapped to genes was generated using HTSeq [25]. For miRNA data, miRDeep2 [26] was performed to verify miRNA specific analysis. In addition, the total reads were first duplicated to the unique transcripts, and then the unique transcripts could be annotated as known miRNA, piRNA, rRNA, tRNA, snRNA, snoRNA, repeat, exon (exon sense and exon antisense), intron (intron sense and intron antisense) and unknown sequences by aligning to miRbase [27], piRBase [28], Rfam [29] and Repbase [30].

Gene expression levels were quantified using reads per kilobase of exon per million reads mapped (FPKM) [31] for mRNA and lncRNA. The values of counts per million (CPM) [29] and transcripts per million (TPM) [32] were preferred to quantify the expression level of miRNA and circRNA respectively.

### Analysis of differentially expressed genes (DEG)

We used the DESeq packages [33] to identify the differentially expressed genes (DEGs) between the samples, and a *p*-value was assigned to each gene to assess its statistical significance. DEGs were defined as those with|log2foldchange|>1 and *p*-value < 0.05. In the following, the normalized count data were used for the downstream analysis of principal components analysis (PCA) and expression correlation matrices.

### Gene Ontology (GO) and Kyoto Encyclopedia of genes and genomes (KEGG) pathway enrichment analyses

GO and KEGG pathway enrichment were performed to reveal the functional annotation of the DEGs. Analyses were based on Metascape [34] (https://metascape.org) and KOBAS [35] (http://bioinfo.org/kobas) software, and *p*-value < 0.05 was considered statistically significant.

### Construction of the protein-protein interaction network

The protein-protein interaction (PPI) network of the DEGs was constructed according to the information in the STRING database [36] (http://string-db.org/). The minimum required interaction score was set at 0.400 and all unconnected nodes were hidden. Subsequently, the sub-network modules of the networks were aggregated and extracted from the PPI network using the Cytoscape plug-in Cytoscape Molecular Complex Detection (MCODE) (http://apps.cytoscape.org/apps/mcode/), and the top 5 regulated genes were selected as the preliminary hub genes according to the order of the topology.

### Construction of ceRNA network

In this study, the differentially expressed RNAs were selected for ceRNA interaction construction. The competing interactions between circRNA-miRNA, lncRNA-miRNA and miRNA-mRNA pairs were predicted based on the databases of MiRanda and PsRobot. The interaction pairs sharing the same miRNAs were identified as candidate miRNA-lncRNA-mRNA, miRNA-circRNA-mRNA interaction network, and miRNA-circRNA-lncRNA-mRNA competing interactions. The predicted ceRNA networks were then visualized using Cytoscape [37].

## Results

### Histological characteristics of the testes

**Fig. 1:**
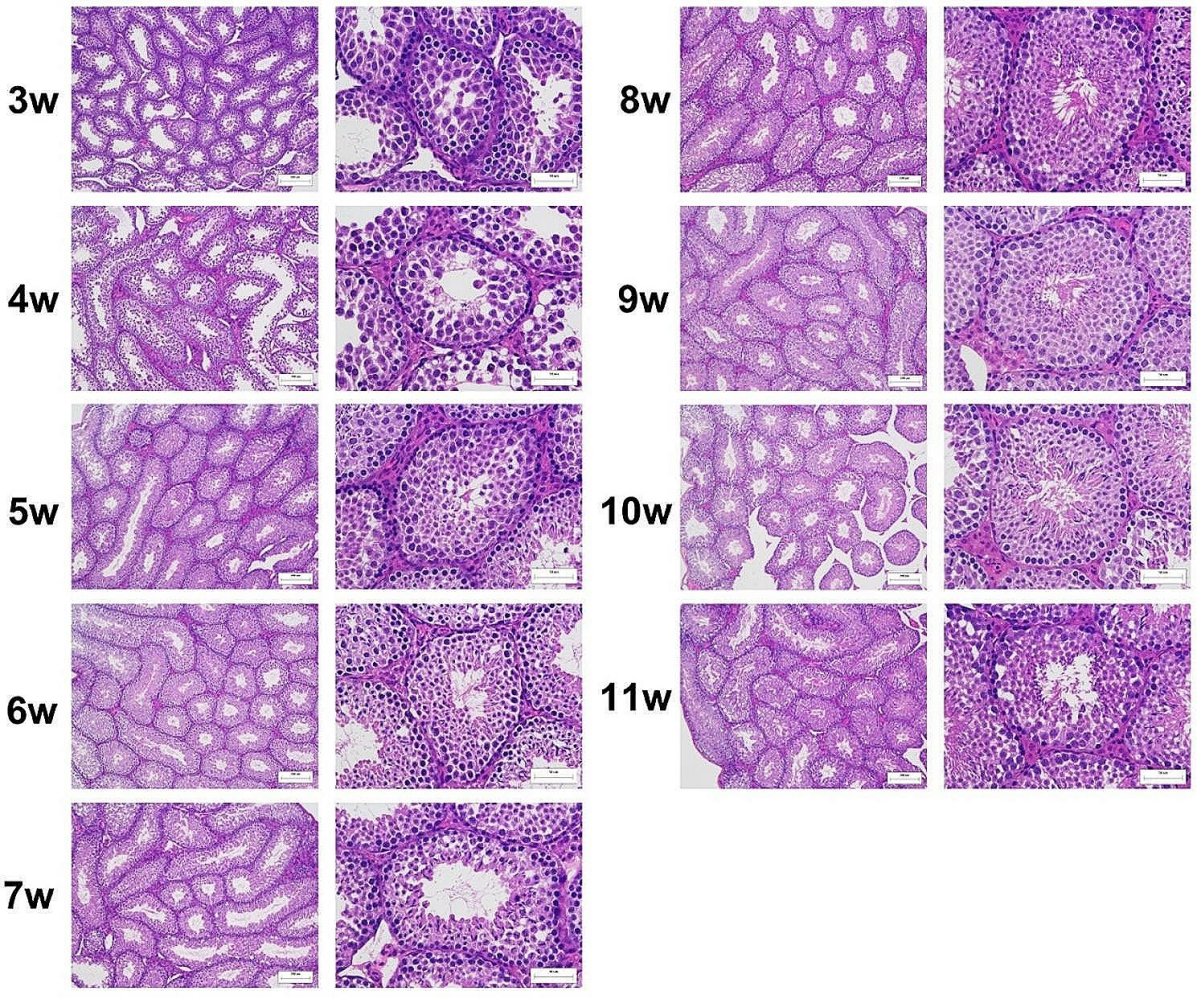
Histology of murine testicular tissues at 3-11w. The left series of micrographs were taken under a microscope at ×100 magnification and the right series at ×400 magnification

Histological analysis of mouse testes showed that the difference between 4w and 5w was obvious under 400x microscopic examination. In the histological sections, no sperm cells could be observed inside the seminiferous tubules of the 3w and 4w testes. However, a few sperms could be detected in the intersecting surface of the testes of 5-week-old mice, and the number of sperm continued to increase over the following weeks. High numbers of sperm cells were observed inside the seminiferous tubules of 8w mice, and there was no significant difference in the number of sperm in the testes of 8-11w mice (Fig. 1).

### Overview of the whole-transcriptome sequencing

**Fig. 2:**
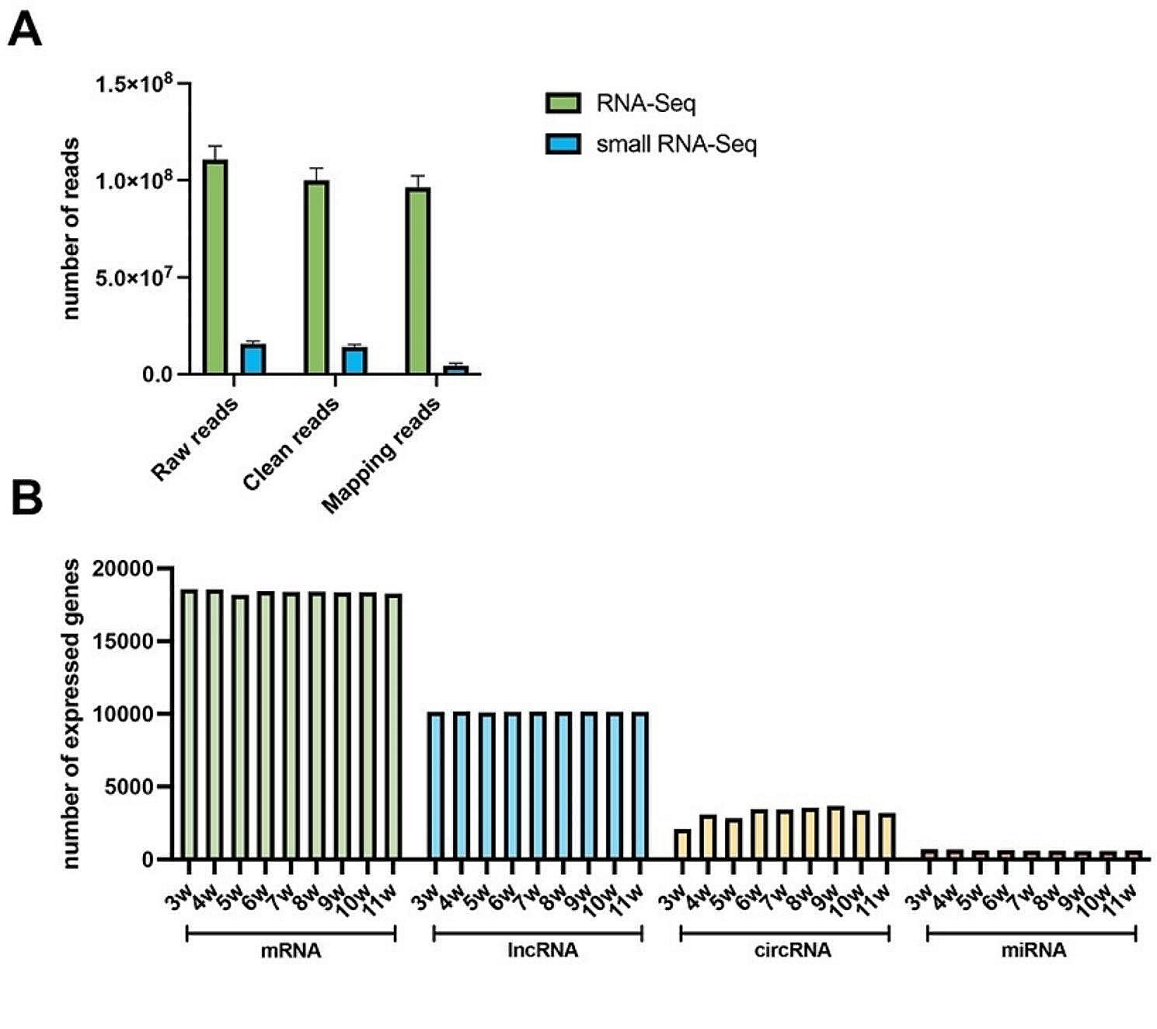
Statistics of whole-transcriptome sequencing. (**A**) Summary of RNA-seq and small RNA-seq statistics. (**B**) Number of genes expressed in the testes of 3-11w mice

In this work, two libraries were constructed to reveal the whole transcriptome profiles in mouse testis. For the RNA-seq libraries, a total of 996,302,214 raw reads were acquired, and 90.32% (899,835,270 out of 996,302,214) of the reads were the clean reads after removing low-quality reads. On average, 96312053.8 clean reads were mapped to the reference genome, resulting in a mapping rate of 96.33%. The small RNA transcriptome profiles were constructed using the small RNA-Seq libraries, which yielded an average of 141,945,984 raw reads and 127,637,426 clean reads (Fig. 2A). The number of the annotated genes is shown in Fig. 2B, and the number of annotated mRNAs and lncRNAs was higher than that of circRNAs and miRNAs. For the RNAs expressed in 3-11w, the comparable expression levels were detected in mRNAs, lncRNAs as well as miRNAs. Fewer miRNAs were found in the testes of 3w and 5w.

### Transcriptomic profile in mouse testis during testicular development

**Fig. 3:**
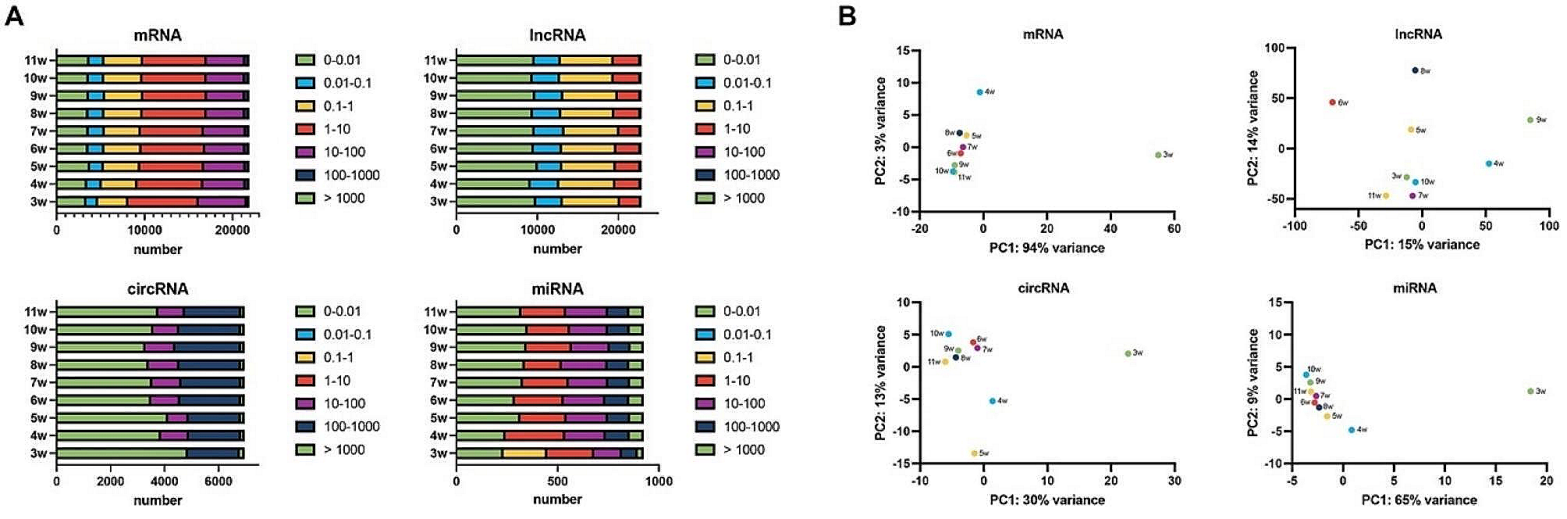
The number of detected transcripts at each postnatal age in seven abundance levels. (**A**) Distribution of different abundances of mRNA, lncRNA, circRNA, and miRNA. (**B**) Principal component analysis (PCA) of mRNA, lncRNA, circRNA, and miRNA datasets

To describe the expression level of the transcripts, all annotated genes were classified into seven categories. The proportion of the RNAs in different categories seemed to be comparable in 3-11w. Compared to the testes at other ages, more mRNAs were expressed at 1–10 FPKM in 3w. The vast majority of the lncRNAs and circRNAs expressed less than 0.01 TPM, and the remaining transcripts were expressed at higher levels in circRNA than in lncRNA. Almost no transcripts expressed > 1,000 FPKM at both mRNA and lncRNA levels. In addition, the circRNAs were absent in the abundances of 0-0.01 TPM, 0.01–0.1 TPM and 1–10 TPM. The circRNAs expressed at 10–100 TPM were missing, and the miRNAs expressed at 0.1-1 CPM were uniquely represented. Although the expression patterns varied during the development, the abundance distributions at 3w were quite specific for circRNA and miRNA (Fig. 3A). The results of the PCA analysis also showed a large difference in 3w from the others. For mRNAs and miRNAs, the expression profiles of 5-11w were clustered in one dimension. The circRNAs of 6-11w were grouped together, while for lncRNAs the expression profiles of different post-natal weeks rarely clustered (Fig. 3B).

### Differential expression analysis

**Fig. 4:**
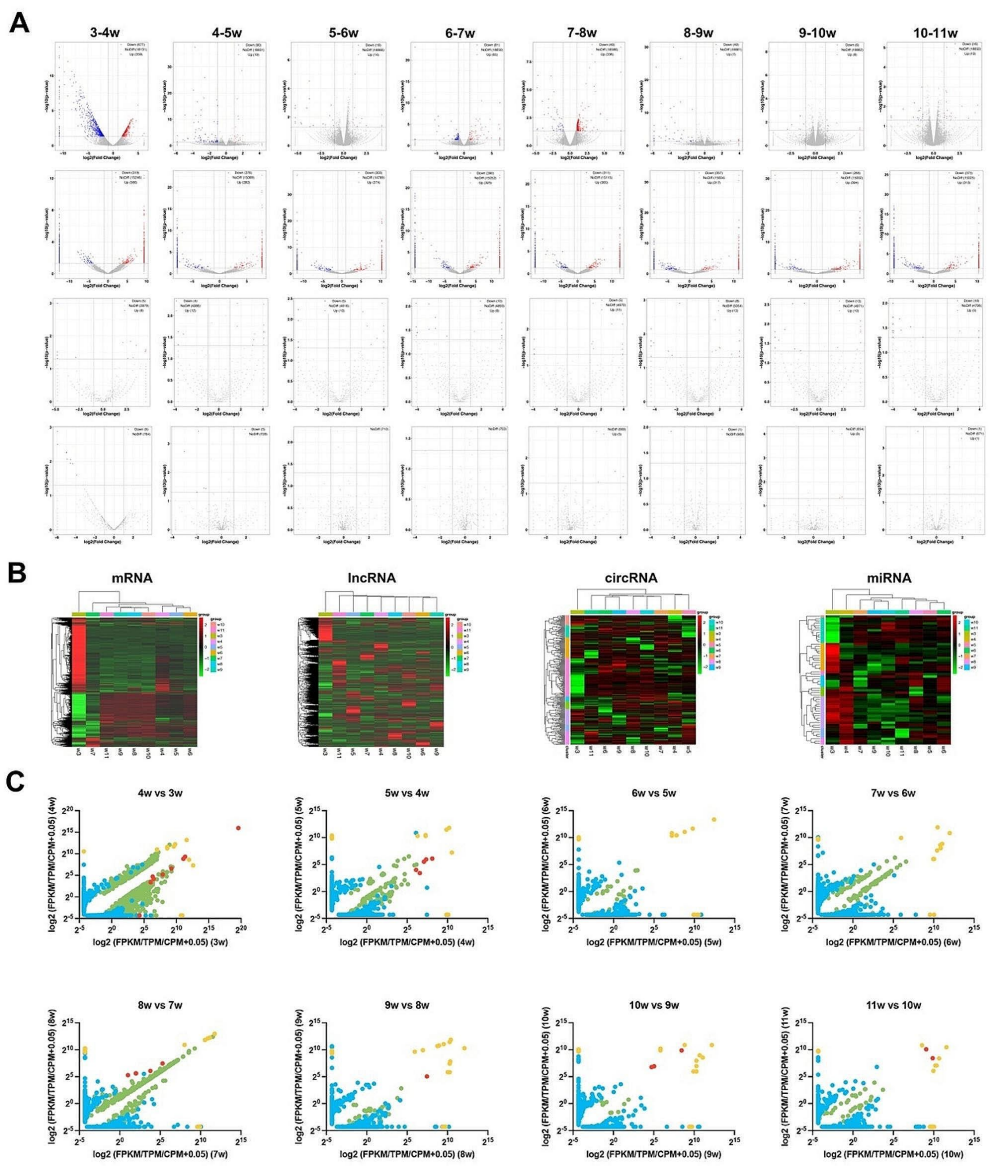
Differential expression analysis of 3-11w testis. (**A**) Volcano plots show significantly differentially expressed genes (from top to bottom: mRNA, lnRNA, miRNA and circRNA). (**B**) Heatmaps of differentially expressed genes. The abscissa represents different samples; the vertical axis represents clusters of DEGs. Red color represents up-regulation; green color represents down-regulation. (**C**) Bubble plots of the DEGs during 3-11w. The x-axis shows log2-transformed expression levels at earlier postnatal weeks and the colors of the bubbles represent the mRNAs (green), lncRNAs (blue), circRNAs (orange) and miRNAs (red)

We identified a total of 7,612 differentially expressed genes (DEGs) in all eight time intervals. The overall cluster analysis of DEGs revealed differences among the samples, especially the profiles of 3w than the others. The most represented RNA type was DElncRNA (5631), followed by DEmRNA (1813), DEcircRNA (143), and DEmiRNA (25). The total number of the up-regulated DEGs and down-regulated DEGs was similar, but DEGs at different developmental stages changed more in mRNA and miRNA. Although continuous DEGs could be observed in lncRNA and circRNA, the number of DElncRNA followed opposite trends to that of the miRNA (Fig. 4A). The clustering heatmaps of DEmRNA, DElncRNA, DEcircRNA, and DEmiRNA were shown in Fig. 3B, which separated the 3w samples from the others, suggesting that a significant difference occurred in the first interval (Fig. 4B).

To further investigate the changes in two adjacent postnatal weeks, we compared the expression levels of the DEGs. For mRNA, most of the DEGs showed a comparative low fold change, especially in the interval of 7-8w. A similar trend was observed in miRNA, which showed higher expression levels. Many datapoints of DEcircRNA and DElncRNA clustered along the x-axis or y-axis, indicating the extreme fold changes in RNA expression (Fig. 4C).

### Dynamic features of testis development related transcripts showing changes from 3 to 11w

**Fig. 5:**
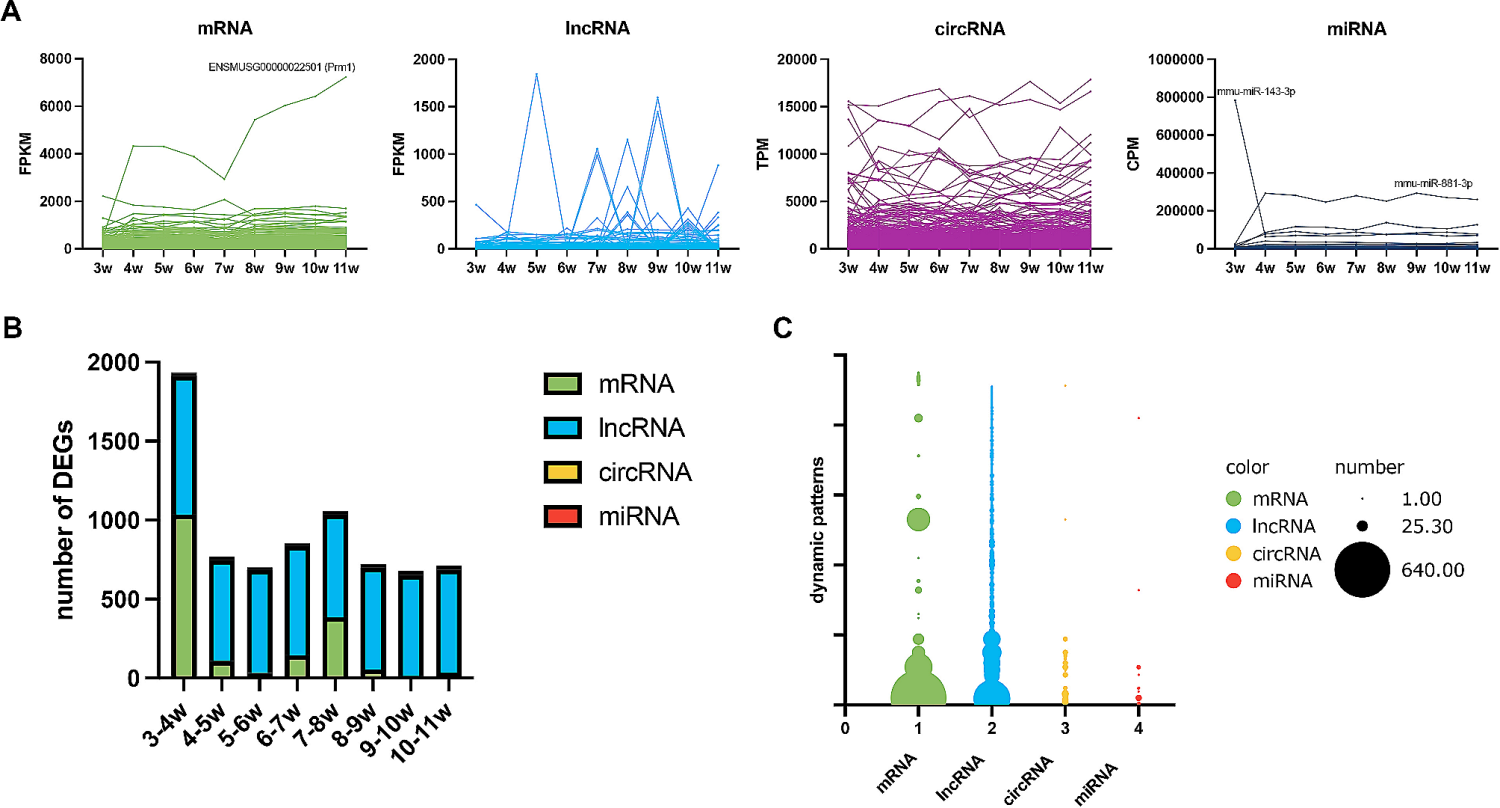
RNA dynamics in 3-11w mouse testis. (**A**) Expression of the RNAs expressed during testis maturation. (**B**) Bar chart of dynamic expression patterns in mRNA, lncRNA, circRNA and miRNA. (**C**) Bubble plots showing the number of DEGs in different classes

To investigate the dynamic expression patterns for each of the genes, the abundance in eight consecutive postnatal ages was analyzed (Fig. 5A). For mRNAs and miRNAs, most of the molecules expressed in a comparably stable manner, and the drastic changes were found in ENSMUSG00000022501 (Prm1), mmu-miR-143-3p, as well as mmu-miR-881-3p. Although the number of drastically altered RNAs was greater in the lncRNAs and circRNAs, many of the expression patterns remained similar.

To further reveal the dynamic expression patterns of the genes, we described the expression pattern between two adjacent postnatal weeks using U (up-regulated expression), D (down-regulated expression) and M (maintained the previous expression status). As a proof of concept, the 3 possible expression states should generate 6,561 (3^8^) possible dynamic expression patterns in the nine developmental stages. However, only a limited number of expression patterns were found regardless of the RNA type. The MMMMMMMM was the most common status, accounting for 93.16% (20,402/21,899), 87.60% (20,022/22,856), 98.31% (6852/6970) and 97.73% (905/926) of the mRNAs, lncRNAs, circRNAs and miRNAs, respectively. A total of 455 unique expression patterns were detected in lncRNAs, which were the most abundant, followed by mRNAs (58), circRNAs (27) and miRNA (9) (Supplementary Material 1 and Table 1). The number of DEGs was more abundant in mRNA and lncRNA when compared to the others (Fig. 5B), and the lncRNAs showed more diversity (Fig. 5C).

**Table 1:** Top 20 dynamic expression patterns in mRNA, lncRNA, circRNA and miRNA

ranking	dynamic status	mRNA (*n* = 21,899)		dynamic status	lncRNA (*n* = 22,856)		dynamic status	circRNA (*n* = 6970)		dynamic status	miRNA (*n* = 926)	
		No.	Perc.		No.	Perc.		No.	Perc.		No.	Perc.
1	MMMMMMMM	20,402	93.16%	MMMMMMMM	20,022	87.60%	MMMMMMMM	6852	98.31%	MMMMMMMM	905	97.73%
2	DMMMMMMM	640	2.92%	UMMMMMMM	280	1.23%	MMMMMUMM	10	0.14%	DMMMMMMM	8	0.86%
3	UMMMMMMM	239	1.09%	DMMMMMMM	161	0.70%	MMMMMMMD	9	0.13%	MMMMUMMM	4	0.43%
4	MMMMUMMM	159	0.73%	MMMMMMMU	104	0.46%	MUMMMMMM	9	0.13%	MDMMMMMM	3	0.32%
5	UMMMUMMM	113	0.52%	MMMMMMMD	92	0.40%	MMMMMMDM	9	0.13%	MMMMMMUM	2	0.22%
6	MDMMMMMM	45	0.21%	MMMMMMUM	80	0.35%	UMMMMMMM	7	0.10%	MMMMMMUD	1	0.11%
7	MMMDMMMM	36	0.16%	MMMDMMMM	76	0.33%	MMMMUMMM	7	0.10%	MMMMMMMU	1	0.11%
8	MMMDUMMM	34	0.16%	MMMUMMMM	63	0.28%	MMMDMMMM	6	0.09%	MDMMUDMM	1	0.11%
9	MMMUDMMM	30	0.14%	MMMMMUMM	63	0.28%	MMMMMDMM	6	0.09%	DDMMMMMM	1	0.11%
10	MMMUMMMM	25	0.11%	MDMMMMMM	60	0.26%	MMUMMMMM	6	0.09%	MMMMMUMM	0	0.00%
11	DDMMMMMM	15	0.07%	MMMMMDMM	60	0.26%	MMMMMMUD	6	0.09%	MMMMMMMD	0	0.00%
12	MMMMDMMM	15	0.07%	MMMDUMMM	57	0.25%	DMMMMMMM	5	0.07%	MUMMMMMM	0	0.00%
13	MMMMMMMD	14	0.06%	MMMUDMMM	55	0.24%	MMMUMMMM	5	0.07%	MMMMMMDM	0	0.00%
14	MMMMMDMM	13	0.06%	MUMMMMMM	55	0.24%	MMMMMMMU	4	0.06%	UMMMMMMM	0	0.00%
15	MMMMMMMU	12	0.05%	MMUMMMMM	54	0.24%	MMMMMMUM	4	0.06%	MMMDMMMM	0	0.00%
16	MMUMMMMM	11	0.05%	MDUMMMMM	54	0.24%	MDMMMMMM	4	0.06%	MMMMMDMM	0	0.00%
17	MDMMUDMM	10	0.05%	MMMMDMMM	53	0.23%	MMMUDMMM	3	0.04%	MMUMMMMM	0	0.00%
18	MUMMMMMM	6	0.03%	MMMMUMMM	52	0.23%	MUDMMMMM	3	0.04%	MMMUMMMM	0	0.00%
19	MMMMMMUM	6	0.03%	MUDMMMMM	52	0.23%	MMUDMMMM	3	0.04%	MMMUDMMM	0	0.00%
20	MMMMMUMM	6	0.03%	MMUDMMMM	52	0.23%	MMMMMUDM	3	0.04%	MUDMMMMM	0	0.00%

### Enrichment and functional annotation analysis of DEmRNAs

**Fig. 6:**
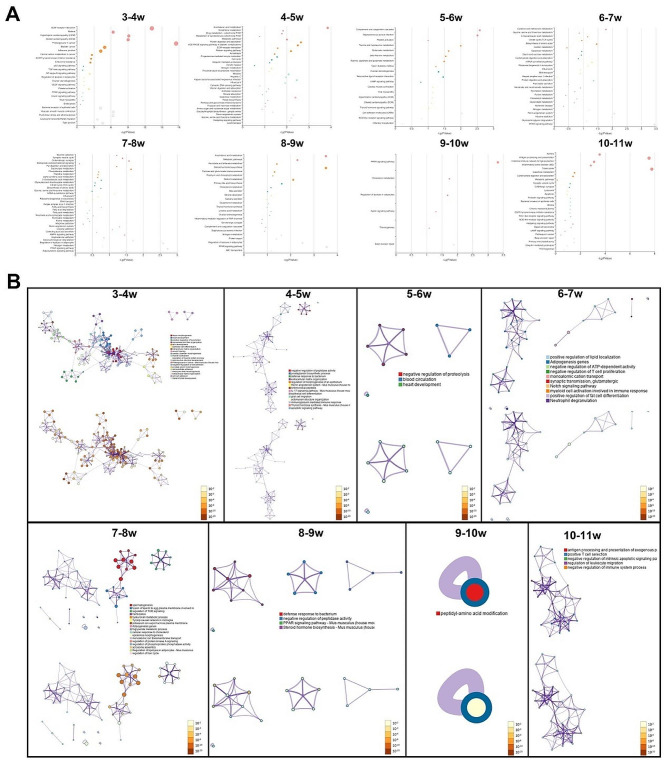
Functional annotation of DEmRNAs at two adjacent timepoints. (**A**) KEGG pathway enrichment analysis and (**B**) GO enrichment analysis enriched by DEGs DEmRNAs. Top: GO terms; Bottom: *p*-values

The GO enrichment and KEGG pathway analyses were performed to reveal the changes in testis maturation. The KEGG pathways results showed that the DEmRNAs were significantly enriched in 614 pathways in 3-11w, and most of the pathways were identified in 3-4w and 10-11w (Supplementary Material 2). In 3-4w, the enriched pathways suggested that most of the DEmRNAs were involved in ECM-receptor interaction pathway and proteoglycans in cancer. Arachidonic acid metabolism was the most significantly enriched pathway in 4-5w. In the third time interval, complement and coagulation cascades, staphylococcus aureus infection and cell adhesion molecules (CAMs) were the top 3 pathways identified. PPAR signaling pathway was found to run through the 6-10w. In 10-11w, the DEmRNAs were significantly enriched in tuberculosis and antigen processing and presentation, of which showed higher *p*-value than the others (Fig. 6A).

The GO analysis suggested that DEmRNAs in 3-4w were enriched in tissue morphogenesis, heart development and positive regulation of locomotion. The DEmRNAs in 4-5w were only involved in negative regulation of peptidase activity. No related processes were detected in 5-7w and 8-10w, which was consistent with the observations in the KEGG analyses. The DEmRNAs from the intervals of 7-8w were mainly associated with the process of spermatogenesis, and the DEmRNAs in 10-11w were significantly enriched in the process of antigen processing and presentation of exogenous peptide antigens via MHC class II (Fig. 6B).

**Fig. 7:**
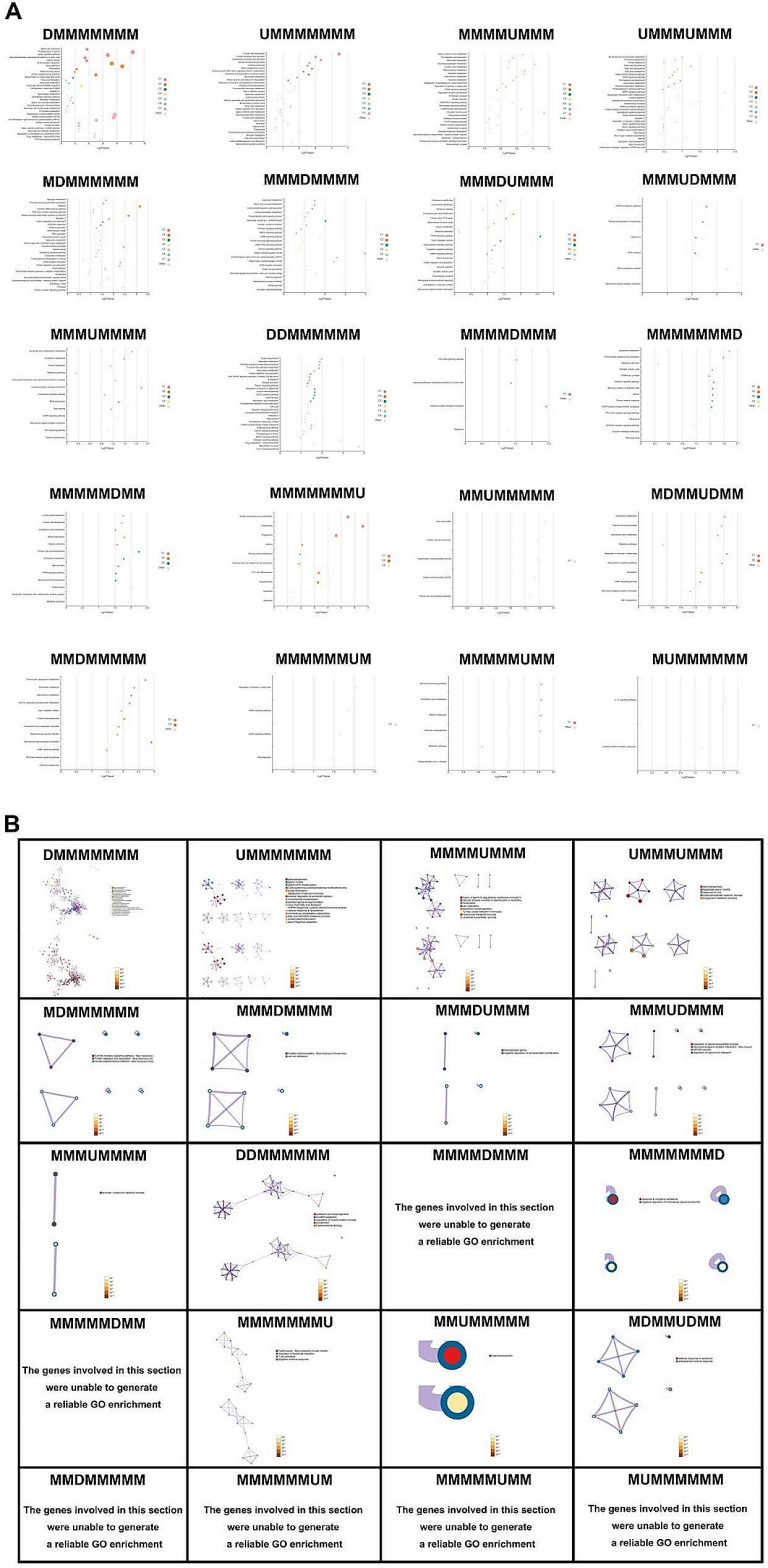
Functional annotation of DEmRNAs in each dynamic pattern. (**A**) KEGG pathway enrichment analysis and (**B**) GO enrichment analysis enriched by DEGs DEmRNAs. Top: GO terms; Bottom: *p*-values

Apart from the pairwise comparisons at two adjacent timepoints, the enrichment analyses were also constructed in different dynamic status. The enrichment analyses require a certain number of genes to generate reliable results. Although there were 59 unique dynamic patterns identified in this study, the outcomes were accessible for merely 20 dynamic patterns (Supplementary Material 3). Most mRNAs expressed differentially only once during 3-11w, and the enrichment results were essentially involved in the results of the adjacent timepoints comparisons. For the rest of the dynamic patterns, the mRNAs in the status of MDMMUDMM were involved in anti-bacterial related response and the pathway of ABC transporters. The PPAR signaling pathway and neuroactive ligand-receptor interaction were the most significant pathways enriched in the mRNAs with MMMDUMMM and MMMUDMMM, respectively. The downregulated DEmRNAs in the first two intervals showed potential function in the epithelial cell morphogenesis (GO: 0003382) and the estrogen signaling pathway. The mRNAs in the status of UMMMUMMM were enriched in the process of spermatogenesis (GO:0007283), which was in concordance to the results observed in 3-4w and 7-8w (Fig. 7A and B).

### Protein-protein interaction (PPI) network and module analysis

**Fig. 8:**
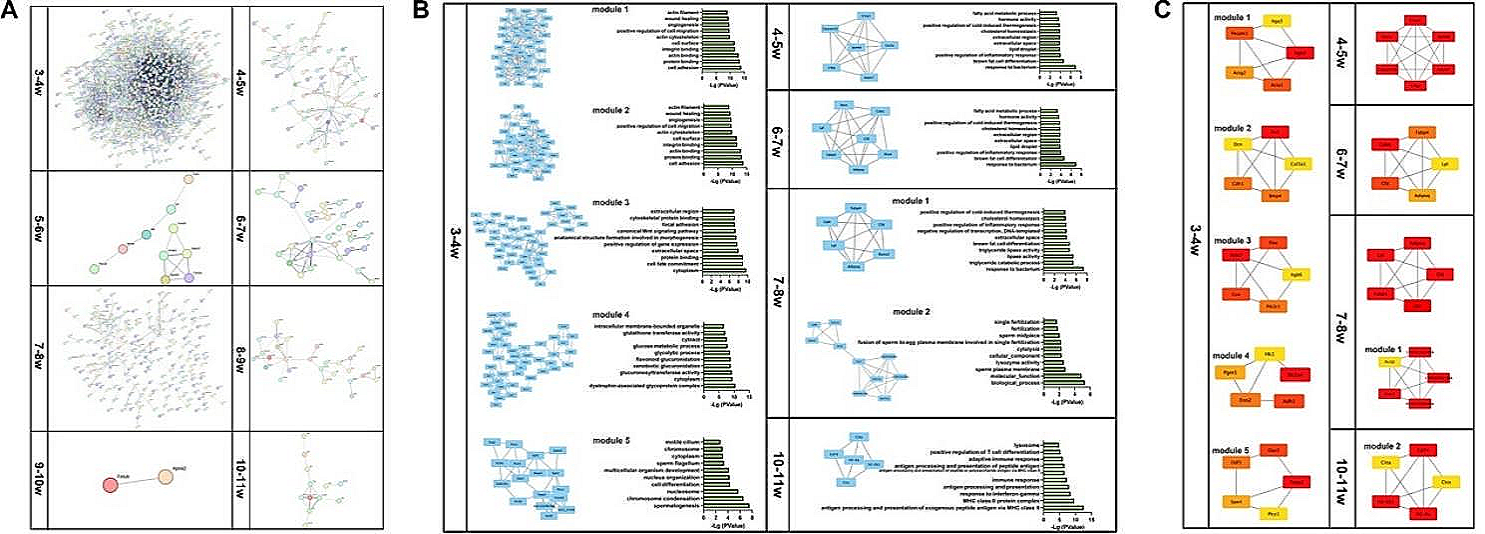
PPI construction, module extraction and hub gene selection. Identification of the hub genes involved in testis development. (**A**) Construction of PPI networks of DEmRNAs. (**B**) Significantly clustered modules of the PPI network and the top 10 GO terms enriched by genes in the modules. (**C**) Hub genes calculated using MCC, DMNC, MNC, degree, EPC, bottleneck, eccentricity, closeness, radiality, betweenness, stress and clustering coefficient algorithms

The STRING database predicted the PPI network of DEmRNAs after eliminating isolated connected nodes. The 3-4w network was the most complex, containing 1,017 nodes and 4,558 edges. The 7-8w network was the second most complex, containing 201 nodes and 265 edges. The 9-10w PPI network, which including 2 nodes and 1 edge, was the simplest in eight intervals (Fig. 8A).

The PPI network of 3-4w was divided into 5 clusters. The first cluster consisted of 39 genes, and they were involved in the process of cell adhesion (GO:0007155), actin binding (GO:0003779), protein binding (GO:0005515), integrin binding (GO:0005178) and cell surface (GO:0009986). The second cluster consisted of 36 genes, which were enriched in the same top five biological processes as the first cluster. The third cluster consisted of 47 genes, and the top five terms were cytoplasm (GO:0005737), cell fate commitment (GO:0045165), protein binding (GO:0005515), extracellular space (GO:0005615) and positive regulation of gene expression (GO:0010628). The fourth cluster was composed of 36 genes enriched in dystrophin-associated glycoprotein complex (GO:0016010), cytoplasm (GO:0005737), glucuronosyltransferase activity (GO:0015020), xenobiotic glucuronidation (GO:0052697) and flavonoid glucuronidation (GO:0052696). The fifth cluster consisted of 36 genes enriched in the process of spermatogenesis (GO:0007283), chromosome condensation (GO:0030261), nucleosome (GO:0000786), cell differentiation (GO:0030154) and nucleus organization (GO:0006997).

One cluster was found in 4-5w and 6-7w. Seven genes formed the only cluster, which was mainly enriched in the process of extracellular region (GO:0005576), sterol esterase activity (GO:0004771), triglyceride lipase activity (GO:0004806), carboxylic ester hydrolase activity (GO:0052689) and flagellated sperm motility (GO:0030317). The module in 6-7w consisted of 7 genes involved in response to bacterium (GO:0009617), brown fat cell differentiation (GO:0050873), positive regulation of inflammatory response (GO:0050729), lipid droplet (GO:0005811) and extracellular space (GO:0005615).

The 7-8w PPI network was divided into 2 clusters. The first cluster contained 6 genes and the second cluster was involved in 10 genes. The genes were involved in the process of response to bacterium (GO:0009617), triglyceride catabolic process (GO:0019433), lipase activity (GO:0016298), triglyceride lipase activity (GO:0004806), and brown fat cell differentiation (GO:0050873).

In 10-11w, the cluster consisted of 5 genes (Ctss, H2-Aa, Cd74, H2-Eb1 and Ciita) involved in a number of immune responses, including antigen processing and presentation of exogenous peptide antigen by MHC class II GO:0019886, MHC class II protein complex GO:0042613, response to interferon-gamma GO:0034341, antigen processing and presentation GO:0019882 (Fig. 8B).

Hub genes were selected based on the top five intersections of all 12 plug-in algorithms (MCC, DMNC, MNC, Degree, EPC, BottleNeck, EcCentricity, Closeness, Radiality, Betweenness, Stress and Clustering Coefficient). The hub genes of module 1 in 3-4w included Itgb3, Pecam1, Actg2, Acta1, Itga3. In module 2, the hub genes were Fn1, Bmp4, Col3a1, Dcn and Cdh1. In module 3, the hub genes were Acta2, Flnc, Gsn, Pik3r1, Itgb6. In module 4, the hub genes were Adh1, Slc2a4, Eno2, Hk1 and Pgm5. Tppp2, Oaz3, Odf1, Plcz1and Spert were the hub genes in the fifth module of 3-4w.

One module was identified in 4-5w and 6-7w, including Spink8, Adam7, Ceacam10, Crisp1, C4bp, Ces5a in 4-5w and Adipoq, Cidec, Cfd, Fabp4 and Lpl in 6-7w. Two modules were found in 7-8w, and the corresponding hub genes were Cfd, Fabp4, Adipoq, Lpl, Lipe and Ccer1, 1700093K21Rik, 1700013G24Rik, 4930505A04Rik, Prr30 respectively. In 10-11w, one module was detected and the hub genes were Cd74, H2-Eb1, H2-Aa, Ciita and Ctss (Fig. 8C).

### Construction of ceRNA networks

**Fig. 9:**
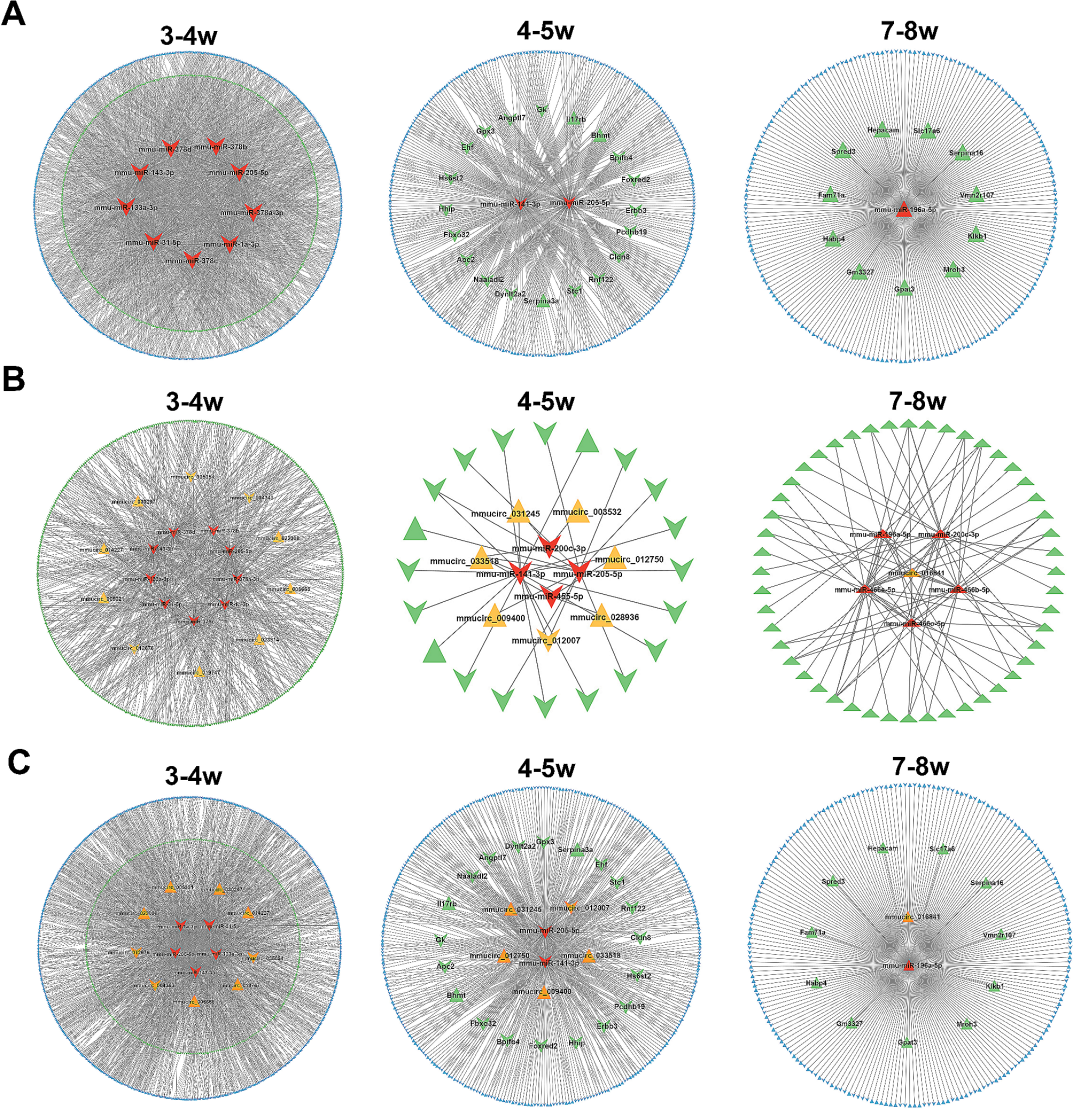
Construction of ceRNA networks in 3-4w, 4-5w and 7-8w. (**A**) The miRNA-lncRNA-mRNA interaction network. (**B**) The miRNA-circRNA-mRNA interaction network. (**C**) The miRNA-circRNA-lncRNA-mRNA interaction network. Blue symbols represent lnRNAs, green symbols represent mRNAs, orange symbols represent circRNAs, and red symbols represent miRNAs. Triangle nodes and arrow nodes indicate the up- and down-regulated expressions, respectively

Based on the competitive endogenous RNA (ceRNA) hypothesis, the lncRNA-miRNA-mRNA networks and circRNA-miRNA-mRNA networks were constructed to explore the functions of lncRNAs and/or circRNAs acting as miRNA sponges in testicular development (Fig. 9A). A total of 1,307 lncRNA-miRNA-mRNA interactions were observed in 3-4w, which were regulated by five miRNAs (mmu-miR-1a-3p, mmu-miR-133a-3p, mmu-miR-205-5p, mmu-miR-143-3p and mmu-miR-31-5p). The lncRNA-miRNA-mRNA ceRNA network in 4-5w contained 431 lncRNA nodes, 21 mRNA nodes, and 452 edges. In 7-8w, the lncRNA-miRNA-mRNA interactions were regulated by mmu-miR-196a-5p and the network contained 222 nodes and 221 edges.

The circRNA-miRNA-mRNA interactions were found in the 3-4w, 4-5w, and 7-8w intervals (Fig. 9B). All nine DEmiRNAs were involved in the circRNA-miRNA-mRNA network of 3-4w. Four miRNAs regulated 34 interaction pairs in 4-5w, including 11 upregulated and 2 downregulated circRNAs, 4 downregulated miRNAs, 3 upregulated and 18 downregulated mRNAs. The circRNA-miRNA-mRNA interactions in 7-8w consisted of 5 upregulated circRNAs, 5 upregulated miRNAs, and 81 upregulated mRNAs.

According to the lncRNA-miRNA-mRNA networks and circRNA-miRNA-mRNA networks, there were DElncRNAs, DEmRNAs and DEcircRNAs regulated by the same DEmiRNA. Furthermore, the genes regulated by the same miRNAs were screened out (Fig. 9C). Five miRNAs (mmu-miR-1a-3p, mmu-miR-133a-3p, mmu-miR-205-5p, mmu-miR-143-3p and mmu-miR-31-5p) were included in the network of 3-4w, involving 69 upregulated mRNAs, 362 downregulated mRNAs, 805 upregulated lncRNAs, 502 downregulated lncRNAs, 4 downregulated circRNAs, and 11 upregulated circRNAs. In the interactions of 4-5w, mmu-miR-141-3p and mmu-miR-205-5p were expressed differentiately, and resulted in 459 interaction pairs. In the interaction network of 7-8w, only one miRNA (mmu-miR-196a-5p) was detected, which was predicted to be regulated by mmucirc_016841.

## Discussion

Male fertility in humans has been decreasing constantly. The reasons for this have not been identified, however, research for the underlying causes will have to focus on spermatogenesis and the organ of gamete production: the testis. Few studies have examined the mouse testis transcriptome, and these have focused on only a few postnatal stages. To date, no multiple transcriptome datasets have been published for such a wide range of postnatal periods. To describe the transcriptome landscape of the developing mouse testis, we collected the testes from 3-week-old to 11-week-old mice, covering the entire period from puberty to adulthood [38]. The morphological characteristics of the testis sections showed the sperm cells occurred inside the seminiferous tubules of 5w, which is in line with general knowledge, suggesting the sample collection was representative and comprehensive. The high clean data rates suggested a reliable dataset for the further analysis. The RNA-seq yielded a total of 899 million reads and the small RNA-seq yielded a total of 127 million reads. The dataset generated over 90% clean reads and the mapping rate of mRNA-seq reached 95%, indicating good reads enrichment. The sequencing metrics were comparable to published studies [39, 40], further demonstrating the reliability of this experiment.

Testicular descent is the most important process in male sexual maturity, and full testicular descent is a two-stage process consisting of transabdominal phase and transinguinal phase [41]. In mice, the first phase begins at 3rd -4th postnatal weeks and the second phase is completed by the end of the 8th postnatal week [42, 43]. Therefore, we hypothesized that the expression profiles in these two phases should be significantly different from those in the later weeks. As expected, the PCA showed a weaker correlation with 3-week-old samples compared to the others in mRNA and miRNA levels, and Pearson’s correlation analysis also showed a weaker correlation with 3-week-old samples compared to the others. Interestingly, the lncRNA clusters separated from each other. It has been reported that lncRNAs regulate multiple gene expressions in both cis- and trans-regulatory role [44], so the difference might be masked by targeting different target genes. Nevertheless, this characteristic was probably used for age estimation.

Testis development is a continuous process involving various molecular events [45]. To reveal the dynamic characteristics in testis development, we identified the DEGs in two consecutive post-natal weeks and listed the patterns in 3-11w. Eight time intervals should theoretically generate 3^8^ possible combinations, but only 0.14% ∼6.93% of the combinations were detected in this study, indicating gene specificity in transcriptomes. Séverine et al. [46] described the miRNA expression profile during early post-natal skeletal muscle growth in mice, and the miRNA expression trends were essentially the same. The lncRNAs and mRNAs in the mouse retina also showed non-random expression status across six developmental stages [47, 48]. Similar results can also been found in developing mouse brain [49] and stomach [50]. Talman et al. [51] collect the mouse ventricular tissue samples on postnatal day 1, 4, 9 and 23, and analyzed them with RNA sequencing. We re-analyzed the data and identified a total of 9 unique dynamic patterns. Considering three time intervals have generated 3^3^ potential combinations, the proportion of detected combinations accounted for 33.33%. This result was higher than our observation, which may be attributed to differences in sampling. Longer sampling intervals would result to a wider range of combinations, which may reduce the probability of occurrence further. Due to the absence of articles at the same time intervals, the properties did not be replicated completely. Nevertheless, based on the observation above, the dynamic patterns did not appear in random. This observation suggested they may fulfill the mission of a specific developing period, then maintain the functional tissue by upholding a certain and stable expression level.

Most genes expressed in the “MMMMMMMM” status, suggesting that the testis maturation process may be driven by some specific genes. For the non-“MMMMMMMM” patterns, the “DMMMMMMM”/“UMMMMMMM” was the most frequently detected in mRNA, lncRNA and miRNA (Fig. 4A and C), suggesting that the genes related to testis development had already been prepared in the 4th post-natal week. Apart from 3 to 4w, that 7-8w was the other turning point in testis development, as mice reach sexual maturity at 8 weeks of age [52]. As expected, “MMMMDMMM”/“MMMMUMMM” ranked third at mRNA and miRNA levels, suggesting that these transcripts may be the key genes driving maturation. Interestingly, not all RNA types (e.g. circRNAs and lncRNAs) followed the mRNA expression trend. Functional proteins consist of complex molecules, and most of them play the biological functions after various post-translational modifications [53]. Therefore, we suspected that the postponed phenotype might be due to the temporal delay in the process of mRNAs to functional proteins. On the other hand, most of the ncRNAs-mRNA interactions were predicted using the bioinformatic methods. With the ongoing research programs, the ncRNA datasets are growing and more favorable answers would be obtained with the improvement of the database. The lncRNA expression patterns were evenly distributed, suggesting the potential for the discovery of age-related biomarkers. Remarkably, ENSMUSG00000022501 (Prm1), mmu-miR-143-3p, and mmu-miR-881-3p showed drastic changes compared to the others, which may reflect the key role they play in male maturation. Protamine 1 (Prm1) has been supposed as a vital protein coding gene in nucleus organization, and it is involved in spermatid development and male fertility [54, 55]. Similarly, mmu-miR-143-3p and mmu-miR-881-3p may also play an indispensable role in maintaining male fertility, but their exact function remains unknown.

Changes in mRNA expression were assumed to have a biological significance as they would be translated directly to the corresponding proteins [56]. To characterize the transcriptional process underlying mouse testis maturation, the KEGG pathway enrichment and GO function annotations of the DEmRNAs were identified. ECM-receptor interaction pathway was the most significantly enriched pathway in 3-4w, consistent with the transcriptome datasets published by Yuan et al.’s [57]. Nevertheless, the process of ECM-derived testicular descent was not fully understood at the time. The subnetwork suggested that glucuronosyltransferase activity (GO:0015020) was involved in 3-4w. UDP-glucuronic acid is the precursor of hyaluronan, which plays a role in human transinguinal testis descent [58]. This observation may indicate changes in the ECM during transinguinal descent via hyaluronic acid metabolism. Recently, Wang et al. [59] compared the ECM components in normal and cryptorchid ziwuling black goat testes and found that the distribution of major ECM components affected the development of seminiferous tubules and Leydig cells. We then constructed the miRNA-lncRNA-mRNA, miRNA-circRNA-mRNA, and miRNA-circRNA-lncRNA-mRNA interaction networks for genes related to the first testicular descent. Nine miRNAs were involved in the ceRNA interactions, but none of them showed associations with testicular development. Sun et al. [60] report that the mice lacking miR-378 have defects in cholesterol homeostasis. In this study, the expression level of cholesterol side-chain cleavage enzyme (CYP11A1) increased from 8.62 FPKM to 52.33 FPKM, so we assumed that differentially expressed miR-378 might provide conditions for the following spermatogenesis by maintaining cholesterol homeostasis. Spermatogenesis was the most striking finding in the fifth post-natal week, and the DEmRNAs in 4-5w were mainly involved in arachidonic acid metabolism. Arachidonic acid has been suggested as a polyunsaturated fatty acid involved in male fertility as early as 1986 [61, 62]. Yu et al. [63] characterized arachidonic acid metabolic changes in asthenozoospermic seminal plasma and found that an abnormal arachidonic acid metabolic network may reduce sperm motility. However, research on this topic has been fragmented and has produced conflicting results [61]. In this study, six hub genes (Adam7, Crisp1, Ceacam10, C4bp, Ces5a and Spink8) and 3 types of ceRNA networks were constructed in 4-5w. The ADAM gene family expresses in reproductive and somatic tissues [64]. Crisp1 and Ceacam10 were found to be the stage-specific marker genes for spermatogenesis [65] and animal maturation [66]. C4bp and Ces5a have also been implicated in male reproduction and rat testis development [67]. Although there was no direct evidence linking Spink8 to testis development, published studies suggested the endopeptidase inhibitor activity of Spink8 [68, 69]. Since seminal endopeptidase is likely to affect sperm motility [70], we hypothesised that it might disturb testicular spermatogenesis via endopeptidase mediated arachidonic acid inhibition. The expression profiles changed continuously over time. From 3w to 5w, the dominant biological process switched from ECM-receptor interaction to arachidonic acid metabolism. At 5-10w, the DEmRNAs were no longer engaged in both pathways, but were all involved in PPAR signaling pathway. PPARs are considered as the nuclear receptors involved in the crosstalk of endocrine signaling pathways through steroid receptors [71]. As androgen plays a vital role in spermatogenesis [72], it is not surprising that the steroidogenesis-related genes were found to be active at these time points. The 8th postnatal week has been considered as the endpoint where testicular descent completes [42], unfortunately the pathways enriched in 7-8w were not sufficient to find out the changes during this particular period. Upregulated mmu-miR-196a-5p increases the number of paraxial mesodermal cells and is implicated in the increased differentiation potential of paraxial mesodermal cells [73]. In this work, mmu-miR-196a-5p was the most involved miRNA and showed associations with the upregulated mRNAs, suggesting the potential roles in promoting the second phase of testicular descent, however, the underlying molecular mechanisms in testicular descent remain to be elucidated. From 8 to 10w, no significant biological process could be enriched, showing the other stationary phase of testicular development. It has been recognized that the testis matures at the end of the 8th postnatal week [42], as expected, no significant changes were observed in the next two weeks. However, DEmRNAs of 10-11w were significantly enriched in the pathway of antigen processing and presentation of exogenous peptide antigen via MHC class II. In vertebrates, the MHC molecules have been recognized for mediating immune responses to foreign antigens [74, 75]. In the testis, MHC class II molecules are involved in spermatogenesis, and appear to produce sperm cells capable of taking up foreign DNA [76]. Guo et al. [77] reported that MHC class II molecules on sperm head and CD4 molecules on oocyte plasma co-mediate intercellular membrane adhesion at the fusion step in fertilization. However, this topic has not been followed up for decades. Recently, the presence of the HLA-DQA1*5 has been strongly associated with overall HLA-DQA1 compatibility in humans and has shown potential as a surrogate marker for assessing overall immunological compatibility in infertile couples [78]. In this study, we also identified H2-Aa, the homologous gene of HLA-DQA1, as one of the hub genes in antigen processing. In addition, H2-Eb1 and CD74 were also identified as the hub genes, suggesting their potential to modulate fertility. However, how the hub genes influence fertilization remained to be elucidated.

## Conclusion

Testicular development is a dynamic and complex process regulated by a number of different gene sets. However, no multiple transcriptome datasets have been published covering such a wide range of postnatal periods. In the present study, we provided a global view of the dynamic transcriptome profiles in 3-11w mice testes, which could track gene expression, molecular networks and differentiation processes during testis maturation. The observations might provide new potential molecular strategies to understand testicular development and maturation process.

### Limitations of the study

The study provided the high-depth data to investigate the dynamic transcriptome profiles during testis maturation, which was the most consecutive periods in testicular developmental research. However, the developmental mechanisms in mouse testis remained the hypotheses, which has not been proved by direct experiments. Without the empirical evidence, we could not directly assign the DEGs to a determinant molecular pathway, hence the research reliability was supported by high quality data and the published practical examples.

### Electronic supplementary material

Below is the link to the electronic supplementary material.

Supplementary Material 1

Supplementary Material 2

Supplementary Material 3

Supplementary Material 4

Supplementary Material 5

Supplementary Material 6

Supplementary Material 7

## Data Availability

The raw sequences were deposited into Sequence Read Archive (SRA) database with the BioProject accession number PRJNA849281 (https://www.ncbi.nlm.nih.gov/sra/PRJNA849281). The expression values were available in Supplementary Material 4 (mRNA), Supplementary Material 5 (lncRNA), Supplementary Material 6 (circRNA) and Supplementary Material 7 (miRNA).
